# Evaluation of Hepatitis C Virus Core Antigen Assay in a Resource-Limited Setting in Pakistan

**DOI:** 10.3390/diagnostics11081354

**Published:** 2021-07-28

**Authors:** Adeel Abid, Murad Uddin, Taj Muhammad, Safia Awan, Tanya Applegate, Gregory J. Dore, Gavin Cloherty, Saeed Hamid

**Affiliations:** 1 Department of Medicine, Aga Khan University, Stadium Road, Karachi 74800, Pakistan; abid.adeel@gmail.com (A.A.); Murad.uddin@aku.edu (M.U.); taj.muhammad@aku.edu (T.M.); safia.awan@aku.edu (S.A.); 2 Kirby Institute, Viral Hepatitis Clinical Research Program, UNSW Sydney, Sydney, NSW 2052, Australia; Tapplegate@kirby.unsw.edu.au (T.A.); gdore@kirby.unsw.edu.au (G.J.D.); 3 Abbott Diagnostics, Abbott Park, IL 60064-3500, USA; gavin.cloherty@abbott.com

**Keywords:** Hepatitis C, HCV Core antigen, GeneXpert® IV, dededecentralized

## Abstract

The diagnosis of Hepatitis C virus (HCV) infection can be challenging due to its cost and a lack of access to centralized testing. There is an urgent need to develop simplified HCV testing algorithms. The aim of this study was to evaluate the performance characteristics of a Hepatitis C core antigen (HCVcAg) assay in a decentralized, resource-limited setting. This is a descriptive cross-sectional study from a highly endemic area of Karachi, Pakistan. Between October 2019 and July 2020, subjects aged 12 years and above who screened positive for HCV antibodies were simultaneously tested for HCV RNA (Xpert HCV Viral Load, GeneXpert^®^ IV, Cepheid, France) and HCVcAg (ARCHITECT HCV Ag assay, Abbott^®^ Diagnostics) to confirm active HCV infection. An Abbott ARCHITECT^®^ i1000SR Immunoassay Analyser was installed at a local district hospital as a point-of-care (POC) facility for HCVcAg testing, while samples for HCV RNA were tested in a central lab. Two hundred individuals (mean age 46.4 ± 14.5 years, 71.5% females), who screened positive for HCV antibody, were included in the study. HCV RNA was detected in 128 (64.0%) while HCVcAg was reactive in 119 (59.5%) cases. Performance of the Immunoassay Analyser was excellent with a higher throughput and quicker readout value compared to the GeneXpert System. The sensitivity and specificity of HCVcAg (≥10 fmol/L) at HCV RNA thresholds of ≥12 was 99.1% (95% CI: 95–100%) and 87.6% (95%CI: 78.4–94%). A strong agreement was observed between the HCVcAg assay and HCV RNA. The ARCHITECT HCV Ag assay showed high sensitivity and specificity compared to HCV RNA in a decentralized, resource-limited setting. It can therefore be used as a confirmatory test in HCV elimination programs, particularly for low-income countries such as Pakistan.

## 1. Introduction

Hepatitis C virus (HCV) infection represents a major public health problem, with approximately 80 million individuals living with HCV around the world resulting in an excess of 350,000 deaths per annum [1,2]. This is of particular importance to people living in remote and resource-limited settings, where low diagnosis and treatment rates are often attributed to additional barriers at several levels including patient, provider, logistical, structural, and health systems [3,4]. Pakistan is a resource-limited country with the second highest burden of HCV infection in the world (i.e., an overall population prevalence of 5%) [5]. Among a population of 208 million [6], an estimated 10 million people have HCV viraemia (i.e., active infection). However, within the country there are several recognized “hot spots” where the prevalence of HCV infection is much higher than the national average. According to a systematic population based seroprevalence survey of HCV in the resource-limited, peri-urban area of Karachi, the HCV antibody positivity was 23.4% [7].

The standard HCV diagnosis is a two-step process. Anti-HCV (HCV antibodies) screening is followed by confirmation of active infection by the detection of a direct marker of viral replication (usually HCV RNA) [8]. However, in most settings the two-step process is expensive and time consuming, as samples need transportation to central lab facilities for HCV RNA detection. This has been shown to increase patient attrition due to the requirement of extra medical visits and the time lag between screening and diagnosis [3]. Thus, there is an urgent need to develop decentralized, simple, and affordable HCV testing methods to identify those with active HCV infection and link them to care as early as possible [9].

HCV core antigen (HCVcAg) is a stable viral protein released during viral assembly and therefore indicates active HCV infection [10]. The Abbott^®^ HCV Ag chemiluminescent micro-particle immunoassay is used to analyze the presence of free circulating HCV core antigen viral particles and bound antibody/antigen complexes [8]. It also quantifies the presence of virus needed for the diagnosis of acute or chronic HCV infection [11]. Analytical sensitivity is the ability of a test to detect very small amounts of a substance, while clinical sensitivity refers to ability of a test to give a positive result if the patient has the disease. With respect to the HCVcAg assay, while previous studies have shown its analytical sensitivity to be inferior to that of nucleic acid testing (NAT), the clinical sensitivity for the detection of active HCV has been seen as comparable between the two assays due to generally high plasma RNA level during active infection [12]. Studies have demonstrated multiple advantages of using HCV core antigen assays over viral molecular testing methods. These include greater affordability, rapid detection, single platform reflex testing to allow for screening and confirmation, multi-analyte testing from one sample with a smaller input volume requirement, and providing sample stability at room temperature, cutting down the need for cold chain transportation [13,14,15]. Furthermore, there is potential to provide an alternative tool for monitoring treatment and post-treatment relapse [16,17].

There is a paucity of data evaluating and validating the use of HCVcAg for diagnosing HCV at a community level, particularly in low and middle income countries (LMICs). The aim of this study was to evaluate the performance characteristics of the Abbott^®^ HCVcAg assay tested in a decentralized location compared to the GeneXpert^®^ HCV Viral Load assay for HCV RNA located in a central lab, using plasma samples from participants of a cohort group living in a resource-limited, HCV endemic area in Pakistan.

## 2. Materials and Methods

### 2.1. Study Design and Participants

This is a descriptive cross-sectional study nested within a larger HCV micro elimination study being conducted in UC-9 and UC-10 of the Malir District, Karachi, Pakistan.

Between 31 October 2019 and 3 July 2020, blood samples of 200 consecutive participants from an ongoing HCV elimination project in two peri-urban union councils (UCs), UC-9 and UC-10 of the Malir District, Karachi, Pakistan, were collected. Study participants were ≥12 years of age, positive for anti-HCV antibodies on a rapid finger stick test, and had given written informed assent or consent for further testing. People ≤12 years of age and those with decompensated liver disease were excluded, as per our community screening protocol. The study protocol was approved by the Ethics Review Committee (ERC) at Aga Khan University, Karachi, Pakistan (ERC Approval number: 2019-0969-3598).

The collected samples were brought to Memon Goth Hospital, Malir district, Karachi (the local community hospital) for testing using the Abbott^®^ HCVcAg assay and the research lab at Aga Khan University, Karachi for Xpert HCV Viral Load assay for HCV RNA.

### 2.2. Decentralized Lab Set-Up

Memon Goth Hospital does not provide testing for HCV RNA or HCVcAg. A make-shift lab was set up at the hospital specially to test HCVcAg as close to the community site as possible to avoid sample transportation, reduce turnaround time for results, and assess the functionality of the Abbott Architect platform in less-than-optimal circumstances.

The Abbott ARCHITECT^®^ i1000SR Immunoassay Analyser was installed in a spare room at the hospital and attached to a 3-volt battery operated emergency power supply, which could run the machine for up to 20 min during a cycle; electricity supply to the hospital was noted to be frequently interrupted due to power outages. The backup power supply was a hospital power generator which supplies power to the whole hospital. The uninterrupted power source (UPS) provided a 20 min margin in which to manually switch the generator on. An air-conditioner was installed in the room to provide reasonable temperatures, as temperatures in this area can exceed 40° C during summer months. A standard refrigerator was placed in the room for sample storage until processing.

### 2.3. Study Procedures

Phlebotomy was undertaken to obtain 10 mL of venous blood in study subjects who screened finger prick HCV antibody positive on Rapid Detection Test (SD Rapid Test 02FK10, Standard Diagnostics, Inc., Republic of Korea). This was conducted by a trained phlebotomist on the same day as when the individual was screened via Rapid Detection Test and the blood was collected for both HCV RNA testing (Xpert HCV Viral load, GeneXpert^®^ IV, Cepheid, France) and HCV core antigen testing (ARCHITECT HCV Ag, Abbott^®^ Diagnostics).

Whole blood was collected in K2-EDTA or SST tubes and kept in Coleman Cooler Box just after collection at 2–8 °C until transportation to the lab within 5–6 h. For HCV RNA testing, samples were transported from the Malir district for 40 km to the research lab at Clinical Trials Unit, Aga Khan University, while for the HCVcAg testing samples were brought to the POC lab at Memon Goth Hospital.

In the two labs, samples for both tests were centrifuged for 15 min at 2000 rpm to separate plasma. After centrifugation and separation, the plasma was held at –20 °C for subsequent testing within the next four to five days.

On the day of testing, specimens were placed at room temperature (20–25 °C) until completely thawed and equilibrated. This was followed by plasma samples being vortexed for 15 s. A minimum of 1 mL plasma was required for the Xpert HCV Viral Load assay while 266 μL of plasma was required for the ARCHITECT HCV Ag assay. The cartridge was subsequently loaded onto the GeneXpert^®^ IV Instrument System or Abbott ARCHITECT^®^ i1000SR Immunoassay Analyser for HCV Viral Load and HCVcAg assays, respectively. Results for both diagnostic tests were interpreted automatically from measured fluorescent signals and embedded calculation algorithms.

### 2.4. Study Assessment

HCV RNA was quantified in plasma samples and tested by (Xpert HCV Viral Load, GeneXpert^®^ IV, Cepheid, France) with the lower limit of quantification being 12 IU/mL. HCV core antigen was quantified in plasma by the two-step chemiluminescent micro-particle immunoassay ARCHITECT HCV Ag on the 128 ARCHITECT i1000SR Immunoassay Analyser (Abbott^®^ Diagnostics). The assay has a cutoff of <3 fmol/L, where samples <3 fmol/L are considered nonreactive for HCV core antigen, ≥3 to ≤10 fmol fall in the ‘grey zone’ and samples ≥10 fmol/L are considered reactive [11].

### 2.5. Statistical Analysis

Means and standard deviations were calculated for continuous data, and number and percentages were calculated for categorical data. The sensitivity and specificity of the HCV core antigen assay (≥3 fmol/L) and (≥10 fmol/L) was evaluated at three different thresholds of HCV RNA: ≥12, ≥1000 IU/mL, and ≥3000 IU/mL. Kappa values were calculated as quantitative measures of agreement between HCV RNA and HCV core antigen assay. Kappa values were interpreted as a value between 0.2 (poor agreement) to 1.0 (perfect agreement). Pearson’s correlation coefficient was used to determine correlation between HCV RNA and HCV core antigen. A Bland–Altman Bias plot was generated to visualize both the agreement and difference between the two assays. Log transformations (i.e., log_10_ of HCVcAg levels (log_10_ fmol/L) and HCV RNA levels (log_10_ IU/mL)) were used in the analysis of correlation and agreement.

All statistical analysis and visualization were carried out using SPSSv22 (IBM, Armonk, NY, USA) and Microsoft Excel. For all statistical purposes, all *p* values were two-sided and a *p*-value of less than 0.05 was considered statistically significant.

## 3. Results

**Figure 1:**
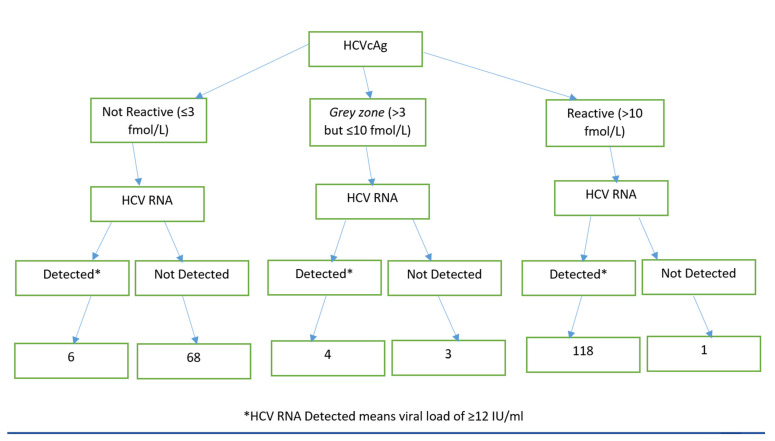
Representation of the results of all study samples (*n* = 200).

The study population consisted of 200 patients, of whom 143 (71.5%) were women. The mean age was 46.4 ± 14.5 years. HCV RNA was detected in 128 (64.0%) participants while HCVcAg assay was reactive in 119 (59.5%) participants; 74 were not reactive and 7 in grey zone (Figure 1). In 6 HCV-RNA positive participants, HCVcAg was not reactive. Conversely, HCVcAg was reactive in 4 participants negative for HCV-RNA (Table 1).

The sensitivity and specificity of the HCV core antigen assay (≥3 fmol/L) at an HCV RNA threshold of ≥12, ≥1000 and ≥3000 IU/ mL is shown in Table 2. Sensitivity of the HCV core antigen assay was comparable with all HCV RNA cut-offs, while specificity was higher with an HCV RNA threshold of ≥3000 IU/ mL (Table 2).

**Figure 2:**
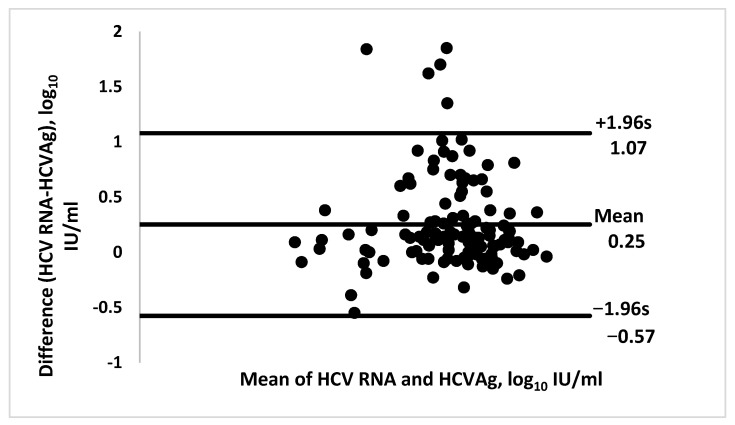
Relationship between the difference in HCV RNA and HCV core antigen (cAg), plotted against the mean of the two measurements in a Bland–Altman plot. The three lines in Figure represent mean of differences-called bias and the other two lines are limits of agreement mean +1.96 SD and mean −1.96 SD. [The x axis represents the logarithmic mean levels of RNA and HCVcAg after conversion of HCVcAg to HCV RNA. The y-axis represents the logarithmic difference of RNA and HCVcAg].

The sensitivity and specificity of HCVcAg (≥10 fmol/L) at an HCV RNA threshold of ≥12, ≥1000, and ≥3000 IU/ mL is shown in Table 3. Sensitivity was the same for all HCV RNA thresholds. However, specificity was higher for the ≥3000 IU/ mL RNA levels. A strong agreement was seen between the HCV core antigen assay (≥3 fmol/L) with HCV RNA, resulting in ĸ values of 0.90, 0.93, and 0.92 for HCV RNA thresholds of ≥12, ≥1000, and ≥3000 IU/mL, respectively. Likewise, a strong agreement was seen between the HCV core antigen assay (≥10fmol/L) with HCV RNA, resulting in ĸ values of 0.88, 0.93 and 0.96 for HCV RNA thresholds of ≥12 IU/mL, ≥1000 IU/mL and ≥3000 IU/mL, respectively (Table 4). Using a conversion factor of 1 fmol/L = 500 IU/mL, the Bland–Altman plot showed the mean bias ±SD between HCV RNA and HCVcAg as 0.25 ± 0.42, and the limits of agreement (95% CI) were −0.576 and 1.078. All axes are represented in logarithmic scale (Figure 2).

## 4. Operational Issues

Electricity supply was not secure due to an average load shedding of 12 h in the area. The uninterrupted power source (UPS) provided a 20 min margin to manually switch the generator on to supply power before the Immunoassay Analyser would stop working and the samples became un-analyzable. On one occasion, 15 samples became unanalyzable due to extended interruption in power supply. Moreover, the Immunoassay Analyser required calibration with each new batch of the test kits, which led to an unforeseen delay of one and half months occurring on one occasion due to shortage of the calibration fluid.

The throughput in one run of testing was two samples in the GeneXpert Instrument System, taking 1 h and 50 min. For the Immunoassay Analyser, the throughput in one run of testing was 65 samples with more than 40 samples being read in 1 h 50 min.

## 5. Discussion

This study evaluated the performance of the HCVcAg assay in a resource-limited community setting against HCV RNA testing in a central lab, using participants enrolled from two peri-urban union councils in Karachi. The HCVcAg assay demonstrated excellent diagnostic performance and strong agreement for both thresholds (≥3 and ≥10 fmol/L) with varying thresholds of HCV RNA. This confirms the suitability of HCVcAg assay for the determination of active HCV infection.

In terms of performance characteristics, the ARCHITECT i1000SR Immunoassay Analyser faced difficulty working at its optimum level during summer months. This was mainly due to unstable electricity which is a problem in Pakistan, being an energy-deficient country with a shortfall in power supply resulting in load shedding [18]. Load shedding is longer in peri-urban and more remote settings in the country like the Malir district, especially in summer. However, with the help of UPS and a generator this problem was managed well throughout the length of the study. Hence, the performance of the Immunoassay Analyser remained excellent with a higher throughput and a quicker readout value being noted compared to GeneXpert Instrument System.

At a threshold of ≥3 fmol/L with HCV RNA limit being ≥12 IU/mL, the sensitivity and specificity was 96.8% and 91.8% (95%CI: 83–97%), respectively. Similarly, the sensitivity and specificity of HCVcAg (≥10 fmol/L) at an HCV RNA limit of ≥12 IU/mL was 99.1% (95% CI: 95–100%) and 87.6% (95% CI: 78.4–94%), respectively. This was comparable to two previous studies conducted in central laboratory settings in Australia, each using the two different thresholds for HCVcAg [3,8]. Additionally, in a bivariate analysis by Freiman et al., the pooled sensitivity and specificity was 93.4% (95% CI: 90.1–96.4%) and 98.8% (95% CI: 90.1–96.4%), respectively [12], and a retrospective large screening cohort by Van Tilborg et al. demonstrated a sensitivity of 94% (95% CI: 86–98%) with a specificity of 100% (95% CI: 94–100%) [14].

Moreover, a very high degree of agreement between HCVcAg assay and HCV RNA was observed. Kappa values for the two tests at both thresholds were very high, in line with the study by Alonso et al. [19]. The Bland–Altman plot showed the mean bias ±SD between HCV RNA and HCVcAg as 0.25 ± 0.42 and the limits of agreement (95% CI) being −0.576 and 1.078. This measurement of agreement was comparable to studies in Australia [10,20]. Hence the data builds on previous recommendations for HCVcAg to be used as an alternative to HCV nucleic acid testing (NAT) for diagnosing active infection [21,22].

This study also shows the feasibility for the HCVcAg assay as a near-POC test for confirmation of active HCV at a community level in a relatively remote setting. This strategy can be adopted in mass elimination programs in developing countries with endemic populations. In LMICs, testing remains inadequate in many settings with high cost of HCV diagnostics and poor infrastructure, inadequate transport systems, insufficient staffing, and limited NAT facilities [8]. The current worldwide testing algorithm for HCV clinical diagnosis requires antibody testing to determine exposure and an RNA test to confirm active infection. The use of HCVcAg as a replacement for NAT (and possibly anti-HCV antibodies) within the LMIC diagnostic and treatment monitoring paradigm is economically attractive, with testing priced at $8–22 (USD) [17]. A cost-effective analysis evaluating the use of HCVcAg as a confirmatory strategy in Central and Western Africa showed that a two-step POC-based strategy including anti-HCV antibody (HCV-Ab) and HCV-RNA testing had the lowest cost, €8.18 per screened individual [23].

There were some limitations to this study. The sample size was relatively small although it was comparable to other studies evaluating HCVcAg against HCV RNA testing [3,8,21]. HCVcAg utility to monitor response to direct-acting antiviral therapy (SVR) was not evaluated, as has been undertaken previously [17,24,25]. Nevertheless, the novel approach to use different cut-thresholds for HCVcAg across different thresholds of HCV RNA addressed the issue of low-level HCV viral loads often seen in a treatment context.

This study provides useful information for policy makers who are planning HCV elimination strategies in remote settings in LMICs. Looking towards alternatives to HCV RNA testing is the way forward especially with new affordable and decentralized tests, such as POC HCVcAg assays currently in development in collaboration with the Foundation for Innovative New Diagnostics and UNITAID [26]. Furthermore, there are future research implications with more validation studies needed to further evaluate the performance of HCVcAg assay in different settings and populations (e.g., patients given Direct Acting Antiviral therapy, those with a sustained virological response, or those with HIV/HCV co-infection). In particular, more suitable diagnostic algorithms need to be created for HCVcAg results falling in the *grey zone* in mass screening programs using the HCVcAg assay for confirmation of active HCV infection. In future, community-based cohort studies with HCV seronegative control groups comparing the HCVcAg assay and HCV RNA should be conducted to better compare specificities of the two confirmatory tests. Moreover, going forward studies should evaluate the analytical performance of HCVcAg in samples of dried venous blood spots (DBS) apart from only plasma in resource-limited settings.

Lastly, another aspect to consider is that the ARCHITECT is a platform that can be used for a wide number of applications beyond HCV and even infectious disease. This capability could be used to strengthen the general healthcare infrastructure in addition to eliminating HCV.

## 6. Conclusions

The HCVcAg assay demonstrated very high sensitivity and specificity when located in a decentralized laboratory in a highly endemic, resource-limited setting. Therefore, the affordable HCVcAg assay is a potentially useful confirmatory test for large scale HCV elimination programs, particularly in low- income countries like Pakistan who have a high burden of HCV infection.

## Figures and Tables

**Table 1:** Discordant results between of HCVcAg assay (≥ 3 fmol/L) and HCV RNA (≥ 12 IU/mL) (*n* = 10).

	HCVcAg Assay (fmol/L)	HCV RNA (IU/mL)
1	3.08 (Reactive)	Not Detected ^Ω^
2	4.61 (Reactive)	Not Detected ^Ω^
3	3.00 (Reactive)	Not Detected ^Ω^
4	10.44 (Reactive)	Not Detected ^Ω^
5	0.00 (Not Reactive)	41 (Detected)
6	1.68 (Not Reactive)	669 (Detected)
7	2.56 (Not Reactive)	90100 (Detected)
8	1.02 (Not Reactive)	638 (Detected)
9	2.53 (Not Reactive)	2990 (Detected)
10	0.00 (Not Reactive)	411 (Detected)

^Ω^ HCV RNA viral load was unquantifiable.

**Table 2:** Sensitivity and specificity of HCV core antigen plasma (≥3 fmol/L) compared with different HCV RNA thresholds.

	Reactive	Not Reactive	Total	Sensitivity (95% CI)	Specificity (95% CI)
HCV RNA Plasma (≥12 IU/mL)					
Detected	122	6	128	96.8% (92–99%)	91.8% (83–97%)
Undetected	4	68	72		
Total	126	74	200		
HCV RNA Plasma (≥1000 IU/mL)					
Detected	121	2	123	96.0% (91–99%)	97.2% (90–100%)
Undetected	5	72	77		
HCV RNA Plasma (≥3000 IU/mL)					
Detected	119	1	120	94.4% (88–98%)	98.6% (92–100%)
Undetected	7	73	80		

**Table 3:** Sensitivity and specificity of HCV core antigen plasma (≥10 fmol/L) compared with different HCV RNA thresholds.

	Reactive	Not Reactive	Sensitivity (95% CI)	Specificity (95% CI)
HCV RNA Plasma (≥12 IU/mL)				
Detected	118	10	99.1% (95–100%)	87.6% (78.4–94%)
Undetected	1	71		
HCV RNA Plasma (≥1000 IU/mL)				
Detected	118	5	99.1% (95–100%)	93.8% (86–98%)
Undetected	1	76		
HCV RNA Plasma (≥3000 IU/mL)				
Detected	118	2	99.1% (95–100%)	97.5% (91–100%)
Undetected	1	79		

**Table 4:** Diagnostic analysis of HCV core antigen plasma compared with HCV RNA (*n* = 200).

	Reactive	Not Reactive	Observed Agreement	ĸ Statistic
HCV core antigen plasma (≥3 fmol/L)				
HCV RNA Plasma (≥12 IU/mL)				
Detected	122	6	190/200	0.90
Undetected	4	68		
HCV RNA Plasma (≥1000 IU/mL)				
Detected	121	2	193/200	0.93
Undetected	5	72		
HCV RNA Plasma (≥3000 IU/mL)				
Detected	119	1	192/200	0.92
Undetected	7	73		
HCV core antigen plasma (≥10 fmol/L)				
HCV RNA Plasma (≥12 IU/mL)				
Detected	118	10	189/200	0.88
Undetected	1	71		
HCV RNA Plasma (≥1000 IU/mL)				
Detected	118	5	194/200	0.93
Undetected	1	76		
HCV RNA Plasma (≥3000 IU/mL)				
Detected	118	2	197/200	0.96
Undetected	1	79		

## Data Availability

Study data is available in archived files kept at the Department of Medicine, Aga Khan University.
